# A practical laboratory index to predict institutionalization and mortality – an 18-year population-based follow-up study

**DOI:** 10.1186/s12877-021-02077-1

**Published:** 2021-02-25

**Authors:** Elisa Heikkilä, Marika Salminen, Anna Viljanen, Taina Katajamäki, Marja-Kaisa Koivula, Kari Pulkki, Raimo Isoaho, Sirkka-Liisa Kivelä, Matti Viitanen, Minna Löppönen, Tero Vahlberg, Laura Viikari, Kerttu Irjala

**Affiliations:** 1 Department of Clinical Medicine, Faculty of Medicine, Unit of Clinical Chemistry, Turku University, 20521 Turku, Finland; 2 Tykslab, Laboratory Division, Turku University Hospital, Hospital District of Southwest Finland, Turku, Finland; 3 Department of Clinical Medicine, Faculty of Medicine, Unit of Family Medicine, University of Turku and Turku University Hospital, 20014 Turku, Finland; 4 City of Turku, Welfare Division, 20101 Turku, Finland; 5 Municipality of Lieto, Health Care Center, 21420 Lieto, Finland; 6 Faculty of Medicine, Department of Clinical Medicine, Unit of Geriatrics, Turku City Hospital, University of Turku, 20700 Turku, Finland; 7 HUS Diagnostic Center, Helsinki University Hospital, Hospital District of Helsinki and Uusimaa (HUS), 00029 Helsinki, Finland; 8 Diagnostic Center, Clinical Chemistry and Hematology, Helsinki University Hospital and University of Helsinki, 00029 Helsinki, Finland; 9 City of Vaasa, Social and Health Care, 65101 Vaasa, Finland; 10 Faculty of Pharmacy, Division of Social Pharmacy, University of Helsinki, 00014 Helsinki, Finland; 11 City of Raisio, Social and Health Care for Elderly, 21200 Raisio, Finland; 12 Department of Clinical Medicine, Faculty of Medicine, Unit of Biostatistics, University of Turku, Turku, Finland

**Keywords:** Laboratory index, Institutionalization, Mortality, Aged

## Abstract

**Background:**

Previously, several indexes based on a large number of clinical and laboratory tests to predict mortality and frailty have been produced. However, there is still a need for an easily applicable screening tool for every-day clinical practice.

**Methods:**

A prospective study with 10- and 18-year follow-ups. Fourteen common laboratory tests were combined to an index. Cox regression model was used to analyse the association of the laboratory index with institutionalization and mortality.

**Results:**

The mean age of the participants (*n* = 1153) was 73.6 (SD 6.8, range 64.0–100.0) years. Altogether, 151 (14.8%) and 305 (29.9%) subjects were institutionalized and 422 (36.6%) and 806 (69.9%) subjects deceased during the 10- and 18-year follow-ups, respectively. Higher LI (laboratory index) scores predicted increased mortality. Mortality rates increased as LI scores increased both in unadjusted and in age- and gender-adjusted models during both follow-ups. The LI did not significantly predict institutionalization either during the 10- or 18-year follow-ups.

**Conclusions:**

A practical index based on routine laboratory tests can be used to predict mortality among older people. An LI could be automatically counted from routine laboratory results and thus an easily applicable screening instrument in clinical settings.

## Background

Frailty is a syndrome defined as a loss of resources in several domains leading to increased vulnerability to stressors [1–4]. Frailty predicts adverse outcomes such as increased falls, hospitalization, morbidity, dependence, and mortality [2, 3, 5]. Symptoms, signs, diseases, disabilities, medications, or laboratory measurements can be combined in an index to measure frailty [1, 6–8], which is calculated as the proportion of individual’s deficits in relation to the total amount of deficits chosen [1, 9–14]. Frailty indexes (FI) are strongly associated with the risk of death, institutionalization, and worsening health status, especially when at least 30 variables are included although different FIs consider different deficits [1, 13, 15, 16]. FIs show a consistent, sub-maximal limit at about 2/3 of the deficits that are considered [1, 17–19].

Earlier studies have demonstrated that prediction of mortality and other adverse health outcomes can also be based on laboratory data [8, 14, 17, 18, 20, 21]. Many factors, such as various diseases, characterized by increased frequency in the elderly, influence blood-derived biochemical values [22]. The impact of these factors may differ in the elderly compared with younger age groups [22]. Howlett et al. [20] demonstrated that a laboratory data -based index can be used to identify older adults at increased risk of death. In their study, a laboratory-based index and a clinical FI were both independently associated with mortality. In a study with older adults in long-term care facilities, Rockwood et al. [18] found a strong linear relationship with a laboratory-based index and a clinical FI. A laboratory-based index could identify long term care residents at increased risk of death. Blodgett et al. [14] examined associations of a laboratory-based index and adverse health outcomes in adult population and found that higher index scores were associated with poor health outcomes at all ages. In their study, there was a weak correlation between a laboratory-based index and a clinical FI. They suggested that a laboratory-based index could be utilized as an early screening tool to identify deficit accumulation at the cellular and molecular level before they become clinically visible [14, 21]. Subclinical deficits, taken together, even including deficits not individually related to death, have been shown to be related to adverse outcomes of aging and precede clinically evident health deficits [18–21, 23]. A laboratory-based index has also been studied in acutely ill older adults admitted to hospital and could be useful also in an acute setting [24–26].

We have earlier demonstrated that clinical frailty tools are applicable screening instruments among Finnish community-dwelling older people [15, 16]. Frailty was associated with higher mortality according to three different clinical frailty screening tools. Simple and fast clinical frailty tools were found comparable with a multidimensional and time-consuming FI [15].

The aim of this study was to analyse whether a laboratory index based on 14 commonly used laboratory tests can be used to evaluate the risk of institutionalization and mortality among Finnish older people during 10- and 18-year follow-ups.

## Methods

### Study design and population

This study is part of a longitudinal epidemiological study carried out in the municipality of Lieto in south-western Finland [27]. All persons born in or prior to the year 1933 (*n* = 1596) were invited to participate in the baseline examination which was carried out between March 1998 and September 1999. Of those eligible, 63 died before they were examined, and 273 refused or did not respond, leaving 1260 (82%) participants, 533 men and 727 women. They were followed-up for institutionalization and mortality for 18 years.

Participants no longer living in Lieto at the end of 2016 (*n* = 86) were excluded from the present analyses predicting institutionalization, as it was not possible to ascertain whether they were institutionalized in their current municipality or whether they lived at home. Sixty-eight participants were already living in institutional care at the start of the study and were excluded from the institutionalization analyses. Also, participants with missing data of analytes needed for the laboratory index (*n* = 107) were excluded leaving 1019 and 1153 participants for the final study cohort predicting institutionalization and mortality, respectively.

### Measurements

Venous blood samples were obtained with minimal stasis between 8 and 10 am after overnight fast at Lieto Health Center. Fresh samples were analyzed at the Central laboratory of Turku University hospital. All participants were given verbal and written instructions on preparing for the blood sample collection before laboratory visit.

### Mortality

Data from all participants who died before January 2017 were obtained from the Statistics of Finland Causes of Death -registry identified with unique personal identification numbers.

### Institutionalization

Institutionalization was defined as permanent entry into a nursing home of which the data were gathered from the municipality’s electronic patient record system and coded by month and year of entry.

### Laboratory index

In this study we created a laboratory index (LI) comprising fourteen laboratory analytes. The laboratory analytes that constitute the LI and their reference ranges or cut-off values are shown in Table 1. The index is calculated as the proportion of individual’s laboratory test results outside reference ranges in relation to the total amount of analytes tested. In selecting the analytes that construct the LI we included routine laboratory parameters that are readily available, and thus easy to test from all elderly patients also in primary health care.

**Table 1:** Reference ranges used for the analytes in laboratory indices for men and women

	Men	Women
Hemoglobin (g/L)	128–168	117–153
Albumin (g/L)	36.1–47.5	34.8–46.1
Calcium (mmol/L)	2.17–2.47	2.17–2.47
Urate (μmol/L)	180–420	130–340
TSH (mU/L)	0.4–4.5	0.4–4.5
Creatinine (μmol/L)	< 135	< 125
Ferritin (μg/L)	20–240	10–100
CRP (mg/L)	< 3	< 3
Sodium (mmol/l)	136–144	136–144
Potassium (mmol/L)	3.5–4.8	3.5–4.8
Glucose (mmol/L)	4.0–6.4	4.0–6.4
ALT (U/L)	< 50	< 35
ALP (U/L)	< 300	< 300
LDL cholesterol (mmol/L)	< 3.5	< 3.5

Abbreviations: *TSH* thyroid stimulating hormone, *CRP* C-reactive protein, *ALT* alanine aminotransferase, *ALP* alkaline phosphatase, *LDL* low-density lipoprotein

The index was constructed by coding each analyte as either 0 or 1; 0 indicates that the value was within the normal range or cutoff and 1 that the value was above or below the normal range or cut-off. The sum of these values was then divided by the total number of the analytes resulting in a score ranging from 0 to 1 for each individual.

To compare the adverse outcomes of individuals with different LI scores, we divided the participants in five categories (1. LI ≤0.08 [≤ 1 laboratory test result outside reference ranges], 2. LI 0.09–0.14 [2 laboratory test results outside reference ranges], 3. LI 0.15–0.21 [3 laboratory test results outside reference ranges], 4. LI 0.22–0.42 [4 to 5 laboratory test results outside reference ranges], and 5. LI ≥0.43 [≥6 laboratory test results outside reference ranges]). The division in five categories is similar to other studies on laboratory-based indexes.

### Statistical analyses

Hazard ratios (HRs) and their 95% confidence intervals for all-cause mortality and institutionalization were calculated using Cox proportional hazard models. Proportional hazards assumption was tested using Martingale residuals. The follow-up periods were calculated from the baseline measurements to the end of the follow-up period of 10 and 18 years or to the death of the individual. Death was used as a competitive factor in the analyses for institutionalization. Both unadjusted and age- and gender-adjusted analyses were conducted. *P* values less than 0.05 were considered statistically significant. All statistical analyzes were performed using SAS System for Windows, version 9.4 (SAS Institute Inc., Cary, NC, USA).

## Results

### Baseline characteristics

The mean age of the participants was 73.6 years (range 64–100 years). The majority (58%) of the participants were women. More detailed baseline characteristics of 1153 study participants are shown in Table 2.

**Table 2:** Baseline characteristics of study participants (*n* = 1153)

	*n* (%)
Age, mean (SD), range	73.6 (6.8), 64–100
Age	
64–74	720 (62)
75–84	334 (29)
≥ 85	99 (9)
Women	663 (58)
Living alone	344 (30)
Education	
More than basic^a^ or basic	103 (9)
Less than basic	1050 (91)
MMSE ≤26	323 (28)
Body mass index, kg/m^2^	
< 20	68 (6)
20–24.9	317 (28)
25–29.9	498 (43)
30–34.9	206 (18)
≥ 35	61 (5)
Number of prescribed medicines	
< 5	855 (74)
5–7	207 (18)
8–9	66 (6)
≥ 10	25 (2)

^a^Six years of elementary school

### Laboratory index in predicting mortality

Altogether, 422 (36.6%) and 806 (69.9%) subjects deceased during the 10- and 18-year follow-ups, respectively.

**Fig. 1:**
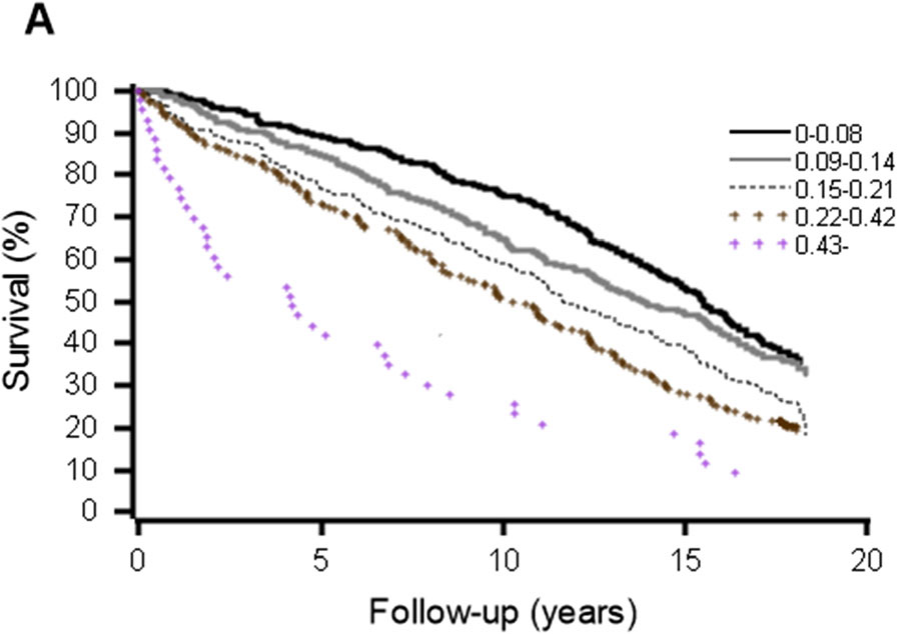
Survival curves by laboratory index (LI) comprising 14 commonly used laboratory tests during the 18-year follow-up. The number of laboratory tests outside reference ranges by categories of LI: 0–0.08 ≤ 1; 0.09–0.14 = 2; 0.15–0.21 = 3; 0.22–0.42 = 4–5; 0.43– ≥ 6

Higher LI predicted increased mortality. Index scores of 0.09 or over and 0.15 or over, predicted increased mortality during the 10- and 18-year follow-ups, respectively. These associations also persisted after adjustments for age and gender. Figure 1 shows Kaplan-Meier survival curves by the categories of LI (Tables 3 and 4).

**Table 3:** Hazard ratios (HR) and their 95% confidence intervals (CI) (in parentheses) of laboratory index (LI) for mortality during the 10-year follow-up

	Total *n*	Deceased *n* (%)	Unadjusted HR (95% CI)	*P*-value	Adjusted^a^ HR (95% CI)	*P*-value
LI						
≤ 0.08^b^	383	92 (24)	1			
0.09–0.14^c^	326	119 (36)	1.61 (1.23–2.12)	< 0.001	1.69 (1.28–2.22)	< 0.001
0.15–0.21^d^	211	87 (41)	1.96 (1.46–2.63)	< 0.001	1.84 (1.37–2.48)	< 0.001
0.22–0.42^e^	190	93 (49)	2.46 (1.84–3.28)	< 0.001	2.20 (1.64–2.94)	< 0.001
≥ 0.43^f^	43	31 (72)	5.56 (3.71–8.40)	< 0.001	3.75 (2.46–5.72)	< 0.001
Total	1153	422 (37)				

^a^Values are adjusted for age and gender

The number of laboratory tests outside reference ranges by categories of LI:

^b^0–0.08 ≤ 1

^c^0.09–0.14 = 2

^d^0.15–0.21 = 3

^e^0.22–0.42 = 4–5

^f^0.43– ≥ 6

**Table 4:** Hazard ratios (HR) and their 95% confidence intervals (CI) (in parentheses) of laboratory index (LI) for mortality during the 18-year follow-up

	Total *n*	Deceased *n* (%)	Unadjusted HR (95% CI)	*P*-value	Adjusted^a^ HR (95% CI)	*P*-value
LI						
≤ 0.08^b^	383	242 (63)	1			
0.09–0.14^c^	326	213 (65)	1.14 (0.95–1.37)	0.169	1.17 (0.98–1.41)	0.091
0.15–0.21^d^	211	160 (76)	1.51 (1.23–1.84)	< 0.001	1.47 (1.20–1.80)	< 0.001
0.22–0.42^e^	190	152 (80)	1.80 (1.47–2.20)	< 0.001	1.72 (1.40–2.12)	< 0.001
≥ 0.43^f^	43	39 (81)	3.41 (2.43–4.79)	< 0.001	2.93 (2.07–4.16)	< 0.001
Total	1153	806 (70)				

^a^Values are adjusted for age and gender

The number of laboratory tests outside reference ranges by categories of LI:

^b^0–0.08 ≤ 1

^c^0.09–0.14 = 2

^d^0.15–0.21 = 3

^e^0.22–0.42 = 4–5

^f^0.43– ≥ 6

### Laboratory index in predicting institutionalization

Altogether, 151 (14.8%) and 305 (29.9%) subjects were institutionalized during the 10- and 18-year follow-ups, respectively. The LI did not significantly predict institutionalization during either of the follow-ups in unadjusted or age- and gender-adjusted models (data not shown).

## Discussion

Our results suggest that an index based on fourteen routine laboratory analytes can be used to predict mortality in an elderly population. The LI was significantly associated with mortality but not with institutionalization during the 10- and 18-year follow-ups. The association of the LI with mortality remained after adjustments for age and gender.

We included fourteen routinely tested laboratory analytes in our index which is a fairly small number of parameters compared to prior studies on laboratory-based indexes [8, 17, 18, 20]. A laboratory index based on smaller number of parameters could be easily applied in use in any hospital or health center. In many countries, laboratory information systems could automatically calculate the LI. In prior studies with more analytes selected in a laboratory index, some analytes reflect the health status of the same or partly the same organ system such as hemoglobin, red blood cells, mean corpuscular volume and hematocrit (hematopoiesis), or alanine aminotransferase, aspartate alanine transferase and gamma-glutamyl transferase (the liver) [17, 20]. In selecting the analytes that construct the LI, care was taken that the information obtained from the analytes did not overlap significantly but captured information with respect to health status of different organ systems.

The LI did not predict institutionalization which seems to be better predicted with clinical FIs [1, 16]. Routine laboratory parameters do not predict dementia or cognitive impairment which are considered the most common causes for institutionalization [28–32]. Other predictive factors for institutionalization, impairing an older person’s ability to live independently, are increased falls, decreasing body mass index [28, 29] and functional impairment and disabilities, especially when combined with cognitive impairment [30–32].

The strengths of our study are the large sample size, good participation rate of 82% and a long follow-up period that enable broad generalizability of the results. The data comes from a community-based representative sample of the Finnish population. The gender distribution of the participants is comparable to the distribution of this age group in the whole country [33], and the prevalence of cognitive impairment is similar to the estimated prevalence in the whole country [34].

A limitation to our study is that the LI has not yet been validated by means of an independent study population or compared to a clinical index. Further research on the validation of the LI is needed.

In clinical settings, the construction of an index using available routine laboratory data may be easier and more harmonized than using data based on clinical assessment. Finding those individuals at an increased risk of death could help clinicians in targeting those patients that need medical interventions. Many of the risk factors that can be identified by laboratory tests, can be treated, when found early enough. As using this laboratory index would not be time-consuming or expensive, it could serve as an alert for the clinician to pay attention to those patients with a high index score. Some studies have found strong and some weak correlations between clinical FIs and laboratory indexes [17, 18, 20, 21, 35]. They seem to be distinct entities although both can be used to predict mortality. Laboratory indexes may find deficits at cellular, molecular or organ level prior to clinical deficits [21]. Previously, there have been some studies that have used both clinical and laboratory data to construct a combined FI, which might assess frailty more accurately [17, 18, 20, 21]. The combination of laboratory and clinical data to construct a frailty index would be an interesting further study also in Finnish elderly population cohort.

## Conclusions

Findings of our study suggest that a practical index based on 14 routine laboratory tests can be used to predict mortality among older people. The number of routine laboratory test results outside reference ranges correlates with older people’s mortality. An LI could be an easily applicable screening instrument in clinical settings.

## Data Availability

The datasets used and/or analysed during the current study are available from the corresponding author on reasonable request.

## Abbreviations

LI: Laboratory index
FI: Frailty index
